# Three new species of *Krogia* (Ramalinaceae, lichenised Ascomycota) from the Paleotropics

**DOI:** 10.3897/mycokeys.40.26025

**Published:** 2018-09-26

**Authors:** Sonja Kistenich, Jouko K. Rikkinen, Holger Thüs, Charles S. Vairappan, Patricia A. Wolseley, Einar Timdal

**Affiliations:** 1 Natural History Museum, University of Oslo, 0318 Oslo, Norway; 2 Finnish Museum of Natural History, University of Helsinki, 00014 Helsinki, Finland; 3 Department of Biosciences, University of Helsinki, 00014 Finland; 4 Department of Life Sciences, The Natural History Museum, London SW75BD, UK; 5 State Museum of Natural History Stuttgart, Rosenstein 1, 70191 Stuttgart, Germany (current address); 6 Institute for Tropical Biology and Conservation, Universiti Malaysia Sabah, 88400 Kota Kinabalu, Sabah, Malaysia

**Keywords:** Borneo, New Caledonia, lichens, *
Phyllopsora
*, phylogeny, rainforest, TLC

## Abstract

*Krogiaborneensis* Kistenich & Timdal, *K.isidiata* Kistenich & Timdal and *K.macrophylla* Kistenich & Timdal are described as new species, the first from Borneo and the two latter from New Caledonia. The new species are supported by morphology, secondary chemistry and DNA sequence data. *Krogiaborneensis* and *K.isidiata* contain sekikaic and homosekikaic acid, both compounds reported here for the first time from the genus. *Krogiamacrophylla* contains an unknown compound apparently related to boninic acid as the major compound. DNA sequences (mtSSU and nrITS) are provided for the first time for *Krogia* and a phylogeny of the genus based on 15 accessions of five of the six accepted species is presented. *Krogiaantillarum* is reported as new to Brazil, Guatemala and Mexico.

## Introduction

*Krogia* Timdal is a corticolous genus occurring in tropical humid forests and rainforests. It closely resembles the much more common genus *Phyllopsora* Müll. Arg. in thallus morphology, but differs mainly in having a weak or absent amyloid reaction in the tholus of the asci and filiform, curved ascospores that are spirally arranged in the ascus (Timdal 2002). In *Phyllopsora*, the tholus shows a deeply amyloid conical structure (*Bacidia*-type) and the ascospores vary from ellipsoid to acicular, but are never spirally arranged. Nearly every examined specimen of *Krogia* has at least some scattered red or purple patches on the thallus or apothecia caused by non-crystalline, acetone-insoluble pigment(s).

Three species of *Krogia* are known: *K.antillarum* Timdal (the West Indies; Timdal 2009), *K.coralloides* Timdal (Mauritius; Timdal 2002) and *K.microphylla* Timdal (the Dominican Republic; Lumbsch et al. 2011). All species are recently discovered and known from only a few collections.

During revision of material of *Phyllopsora* from Southeast Asia and Oceania, we have come across material of three apparently undescribed *Krogia* species. There are no published sequences of *Krogia*, but we have provided sequences of the mitochondrial small subunit (mtSSU) and of the nuclear ribosomal transcribed spacer region (ITS) from the three putative new species and from two of the three previously described species. The sequences, some of which were taken from an unpublished paper on the phylogeny of the Ramalinaceae (Kistenich et al. in press), were used to infer a phylogeny.

## Material and methods

### The specimens

The specimens of the three new species were discovered during ongoing global studies of *Phyllopsora* by Kistenich and Timdal in material provided by Rikkinen (New Caledonia) and Thüs, Vairappan and Wolseley (Borneo), with additional specimens provided by A. Elvebakk (New Caledonia) and A. Paukov (Borneo). The specimens are deposited in B, BM, BORH, H, O and PC. DNA sequences of the two previously described *Krogia* species were generated from specimens in B and O and from a specimen provided by P. Diederich (hb Diederich). Additionally, we included 14 mtSSU and 12 ITS sequences (Table 1) from nine species in six genera known to be closely related to *Krogia* as well as from the holotype of the genus *Krogia*, *K.coralloides*, from a previous molecular study on the family Ramalinaceae (Kistenich et al. in press).

**Table 1.** Specimens used in this study with voucher information, major lichen substances and GenBank accession numbers. New sequences are indicated by accession numbers in bold.

Species and sequence ID	Voucher	Major lichen substances	mtSSU	ITS
* Aciculopsora salmonea *	Costa Rica, 2004, Lücking 17543 (BR), **isotype**	–	MG925842	MG925948
* Bacidia rosella *	Sweden, 1997, Ekman 3117 (BG)	–	AY300877	AF282086
* Bacidia rubella *	Switzerland, van den Boom 41103 (LG DNA 578)	–	JQ796830	JQ796852
* Bacidia sipmanii *	Tenerife, Sérusiaux s.n. (LG DNA 361)	–	JQ796832	JQ796853
* Bacidina brittoniana *	USA, 1999, Ekman 3657 (BG)	–	–	MG925954
* Bacidina delicata *	France, Sérusiaux s. n. (LG DNA 369)	–	JQ796834	JQ796854
* Bacidina neosquamulosa *	Netherlands, van den Boom 41056 (LG DNA 490)	–	JQ796837	JQ796855
* Bacidina phacodes *	Sweden, 1998, Ekman 3414 (UPS)	–	AY567725	AF282100
*Eschatogoniaprolifera* I	Peru, 2006, Timdal 10207 (O)	didymic acid	MG925870	MG925969
*Eschatogoniaprolifera* II	Peru, 2006, Timdal 10429 (O)	didymic acid	MG925871	MG925970
*Krogiaantillarum* I	Trinidad And Tobago, 2008, Rui & Timdal 10844 (O), **paratype**	4-O-methylcrypto-chlorophaeic acid	**MH174271**	**MH174281**
*Krogiaantillarum* II	Guatemala, 2002, Andersohn s.n. (B)	4-O-methylcrypto-chlorophaeic acid	**MH174272**	–
*Krogiaantillarum* III	Mexico, 1994, Wolf & Sipman 2052 (B)	4-O-methylcrypto-chlorophaeic acid	**MH174273**	**MH174282**
*Krogiaantillarum* IV	Brazil, 2015, Dahl, Kistenich, Timdal & Toreskaas AM-39 (O)	4-O-methylcrypto-chlorophaeic acid	**MH174274**	**MH174283**
*Krogiaborneensis* I	Malaysia, 2013, Vairappan & Thüs L291 (BORH), **holotype**	sekikaic acid, homosekikaic acid	**MH174275**	–
*Krogiaborneensis* II	Malaysia, 2012, Wolseley, Thüs & Vairappan D-3-10-2 (BM)	sekikaic acid, homosekikaic acid	**MH174276**	–
*Krogiaborneensis* III	Malaysia, 2014, Paukov 2234 (B)	sekikaic acid, homosekikaic acid	**MH174277**	–
* Krogia borneensis *	Malaysia, 1997, Wolseley Q21 p.p. (BM)	sekikaic acid, homosekikaic acid	–	–
* Krogia borneensis *	Malaysia, 2013, Vairappan & Thüs L229 (BM)	sekikaic acid, homosekikaic acid	–	–
*Krogiacoralloides* I	Mauritius, 1991, Krog & Timdal MAU51/83 (O), **holotype**	boninic acid, unknown	MG925875	MG925977
*Krogiacoralloides* II	Mauritius, 2016, Diederich 18455 (hb. Diederich)	boninic acid, unknown	**MH174278**	**MH174284**
*Krogiaisidiata* I	New Caledonia, 2005, Elvebakk 05:633 (O), **holotype**	sekikaic acid, homosekikaic acid	–	**MH174285**
*Krogiaisidiata* II	New Caledonia, 2016, Rikkinen 34385 (H)	sekikaic acid, homosekikaic acid	**MH174279**	**MH174286**
* Krogia isidiata *	New Caledonia, 2016, Rikkinen 35034 (H)	sekikaic acid, homosekikaic acid	–	–
* Krogia isidiata *	New Caledonia, 2016, Rikkinen 35688 (H)	sekikaic acid, homosekikaic acid	–	–
*Krogiamacrophylla* I	New Caledonia, 2016, Rikkinen 36047 (H)	unknown	–	**MH174287**
*Krogiamacrophylla* II	New Caledonia, 2016, Rikkinen 36077 (H), **holotype**	unknown	–	**MH174288**
*Krogiamacrophylla* III	New Caledonia, 2016, Rikkinen 35037 (H)	unknown	–	**MH174289**
*Krogiamacrophylla* IV	New Caledonia, 2011, Rikkinen 38565 (H)	unknown	**MH174280**	**MH174290**
*Physcidiawrightii* I	Mauritius, 1991, Krog & Timdal MAU14/14 (O)	sekikaic acid, divaricatic acid	MG925911	–
*Physcidiawrightii* II	Mauritius, 1991, Krog & Timdal MAU13/10 (O)	sekikaic acid, divaricatic acid	MG925912	–
* Toninia cinereovirens *	Norway, 1994, Haugan & Timdal 7953 (O)	–	AY567724	AF282104
* Waynea californica *	USA, 1995, Ekman L1486 (UPS)	–	MG925947	–

### Anatomy

Microscope sections were cut using a freezing microtome and mounted in water, 10% KOH (K), lactophenol cotton blue and a modified Lugol’s solution, in which water was replaced by 50% lactic acid. Amyloid reactions were observed in the modified Lugol’s solution after pretreatment in K and crystals of lichen substances were observed using polarised light.

### Secondary chemistry

Thin-layer chromatography (TLC) was performed in accordance with the methods of Culberson (1972), modified by Menlove (1974) and Culberson and Johnson (1982). Examinations were made in the three standard solvent systems A, B' and C.

### DNA extraction, PCR and sequencing

We extracted DNA from apothecia and/or thallus tissue of 14 *Krogia* specimens. The DNA extraction followed the protocol described by Bendiksby and Timdal (2013). We selected the two genetic markers mtSSU and nrITS (including ITS1, 5.8S and ITS2) for molecular analyses. Polymerase chain reactions (PCR) were performed with the primer pairs mtSSU1 and mtSSU3R (Zoller et al. 1999) for mtSSU as well as ITS1-F (Gardes and Bruns 1993) and ITS4 (White et al. 1990) for ITS. In case of poor amplification success, internal primers were used: mtSSUF (5’-ACCAGTAGTGAAGTATGTTGTT-3’) and mtSSUR (5’-AACAACATACTTCACTACTGGT-3’) for mtSSU and ITS_lichF and ITS_lichR (Bendiksby and Timdal 2013) for ITS. We used the following cycling conditions: 95 °C for 7 min, 35 cycles of 95 °C for 30 s, 60 °C for 30 s, 72 °C for 30 s, followed by 72 °C for 7 min. We used Illustra PuReTaq Ready-To-Go PCR Beads (GE Healthcare, Buckinghamshire, UK) with half-sized reactions, i.e. prior to adding DNA, we transferred 12 µl of the mixture to a new PCR tube. To this, we added 0.5 µl of template DNA and 1 µl of each primer (10 µM). The PCR products were purified with the Illustra ExoProStar Clean-Up Kit (GE Healthcare, Buckinghamshire, UK) following the manufacturer’s instructions, but with a 10-fold enzyme dilution. We sent the purified PCR products to Macrogen Europe (Amsterdam, The Netherlands) for Sanger sequencing according to the company’s instructions for sample preparation.

### DNA sequence analysis

We assembled and edited the resulting sequences using the software Geneious R9 (Kearse et al. 2012). For the separate alignment of the variable ITS1 and ITS2 sequences, we used PASTA version 1.7 (Mirarab et al. 2015) with OPAL as aligner and merger, the maximum subproblem set to 50%, RAxML as the tree estimator under a GTR+Γ model and a maximum of 500 iterations. We also used PASTA for the mtSSU alignment with the same settings except that we used a GTR+I+Γ model. As the 5.8S alignment contains mainly conserved regions, the online version of MAFFT version 7.313 (http://mafft.cbrc.jp/alignment/software/; Katoh and Standley 2013) was used (G-INS-i) with default settings except that the scoring matrix was set to 2PAM. Alignments were concatenated for subsequent analyses.

We used PartitionFinder2 (Lanfear et al. 2016) to infer the best-fitting substitution models and partitioning scheme for the concatenated alignment with the Bayesian Information Criterion (BIC) to select amongst all possible combinations of models implemented in MrBayes (1-, 2- and 6-rate models). Subset rates were treated as proportional (‘linked branch lengths’). We defined four potential subsets prior to the analysis: mtSSU, ITS1, 5.8S and ITS2.

Three *Bacidia* De Not. species, *B.rosella* (Pers.) De Not., *B.rubella* (Hoffm.) A. Massal. and *B.sipmanii* M. Brand et al., were used as outgroup in all phylogenetic analyses based on the molecular phylogeny of the Ramalinaceae (Kistenich et al. in press). We checked for incompatibilities amongst gene trees by subjecting each marker to a simple maximum likelihood bootstrap analysis as implemented in RAxML Black Box 8.2.10 (Stamatakis 2014) on the CIPRES webserver (Miller et al. 2010) with default settings. Resulting gene trees were inspected manually for incompatibilities.

The alignment was subjected to maximum likelihood analyses using Garli 2.01 (Zwickl 2006) on the CIPRES webserver (Miller et al. 2010) and on the Abel high performance computing cluster (University of Oslo, Norway) under the models and partitioning scheme suggested by PartitionFinder2. We searched for the best tree using 500 repetitions from a random tree. We ran the nonparametric bootstrapping analysis with 500 replicates, each on 10 search replicates from a random tree.

We analysed the alignment phylogenetically using MrBayes 3.2.6 (Altekar et al. 2004; Ronquist and Huelsenbeck 2003) with BEAGLE (Ayres et al. 2012) on the CIPRES webserver (Miller et al. 2010). We used a (1, 1, 1, 1, 1, 1) Dirichlet for the rate matrix, a (1, 1, 1, 1) Dirichlet for the state frequencies, an exponential (1) distribution for the gamma shape parameter and a uniform (0, 1) distribution for the proportion of invariable sites. Subset rates were assumed proportional with the prior distribution following a (1, 1, 1, 1, 1, 1, 1) Dirichlet. We assumed a compound Dirichlet prior on branch lengths (Rannala et al. 2011; Zhang et al. 2012). For the gamma distribution component of this prior, we set α = 1 and β = 0.5, as the expected tree length α/β (taken from the preceding maximum likelihood analysis) was approximately 1.9. The Dirichlet component of the distribution was set to the default (1, 1). Four parallel Markov chain Monte Carlo (MCMC) runs were performed, each with six chains and the temperature increment parameter set to 0.2 (Altekar et al. 2004). The appropriate degree of heating, adjusted for swap rates in the interval 0.1–0.7, was determined by monitoring cold and hot chains in preliminary runs. We used a burnin of 50% and sampled every 1000^th^ tree. The runs were diagnosed for convergence every 10^6^ generations and were set to terminate either at convergence or after having reached 100×10^6^ generations. Convergence was defined as an average standard deviation of split frequencies (ASDSF) smaller than 0.01. We projected the bootstrap support (BS) values from the Garli-analysis on to the MrBayes majority rule consensus tree with posterior probabilities (PP) and collapsed branches with BS < 50 and PP < 0.7. The resulting trees were edited in TreeGraph 2 (Stöver and Müller 2010).

## Results

### Secondary chemistry

The results of the TLC analyses are shown in Table 1. We identified four lichen substances: 4-O-methylcryptochlorophaeic acid (in *K.antillarum*), sekikaic acid and homosekikaic acid (in *K.borneensis* and *K.isidiata*) and boninic acid (in *K.coralloides*). An unidentified major compound, similar to boninic acid in colour and fluorescence on the developed chromatograms, occurred in *K.coralloides* and *K.macrophylla*. On the chromatograms, the two compounds were first pale brown, then after a few days turning greyish-pink, UV_366_+ blue and occurred in R_f_-classes A:5, B':5, C:6; the unknown moved just above boninic acid in all solvent systems.

### Molecular data and phylogenetic analyses

We successfully generated DNA sequences for 14 *Krogia* specimens, including 10 mtSSU and 10 ITS sequences (Table 1). The final dataset comprised 29 accessions (Table 1) and resulted in a 1424 bp long alignment counting 28% missing data and 470 parsimony-informative sites. The alignment is available at TreeBase (https://treebase.org – study no. 22518).

**Figure 1.**
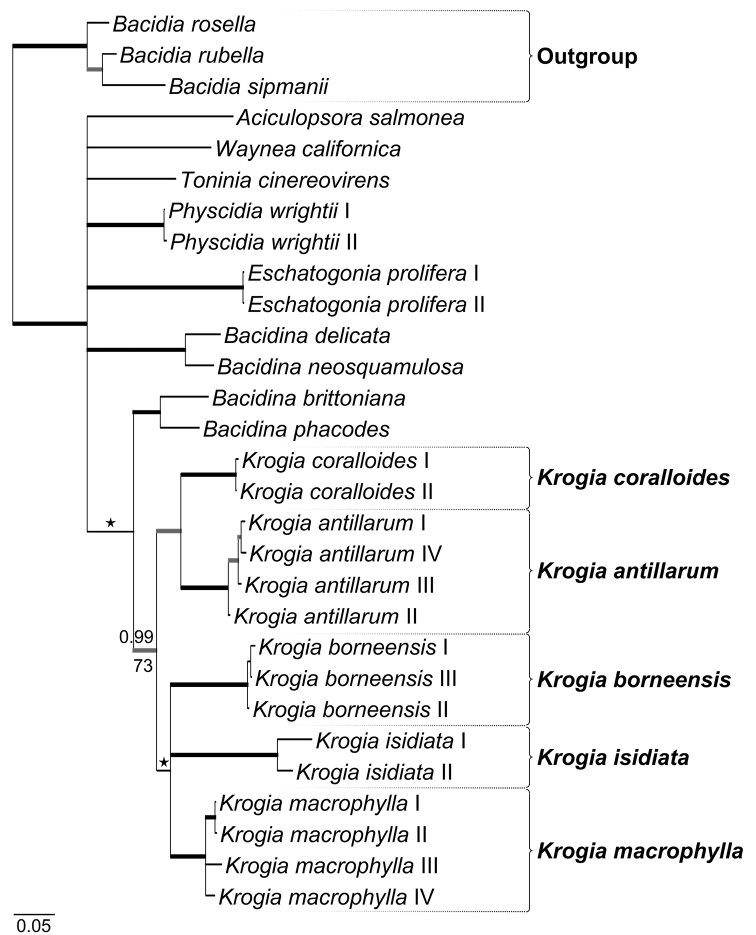
Hypothesis of the phylogenetic relationships and placement of the 15 *Krogia* accessions. It shows the extended majority-rule consensus tree resulting from the Bayesian MCMC analysis with Bayesian PP ≥ 0.7 (above branch) and/or Garli maximum likelihood BS ≥ 50 (below branch) and branch lengths. Strongly supported branches (PP ≥ 0.95 and BS ≥ 95) are marked in bold; branches with PP ≥ 0.95 and BS ≥ 70 are marked in bold grey; branches only supported by PP ≥ 0.7 are marked with an asterisk above the branch. *Bacidiarosella*, *B.rubella* and *B.sipmanii* were used as outgroup. Scale bar indicates 0.05 changes per site.

Initial RAxML analyses produced congruent gene trees of the mtSSU and ITS datasets; only unsupported (< 0.7) topological differences between the consensus trees were observed. We therefore continued with the subsequent phylogenetic analyses. PartitionFinder2 suggested three subsets and two different substitution models, the GTR+G model for (1) mtSSU, (2) ITS1 and ITS2 and the K80+I model for (3) 5.8S. The likelihood score of the best tree found by Garli was –8023.487881. The Bayesian analysis halted automatically after 3 million generations, when the ASDSF in the last 50% of each run had fallen below 0.01. We used 6004 trees for constructing the final majority-rule consensus tree. The phylogenetic results generated by Garli and MrBayes showed no incongruences. The extended majority-rule consensus tree of our alignment (Fig. 1), based on the Bayesian topology with all compatible groups (BS ≥ 50 and/or PP ≥ 0.7), shows that all *Krogia* accessions group together in five distinct and well-supported clades with short terminal branches. Accessions of *Bacidina* Vězda were resolved as the phylogenetic sister clade to the *Krogia* accessions, albeit only supported by PP. Not all *Bacidina* accessions formed a distinct group, but were split in two clades. Except for accessions of the same species, i.e. *Eschatogoniaprolifera* (Mont.) R. Sant. and *Physcidiawrightii* (Tuck.) Tuck., there was poor resolution for the remaining accessions resulting in polytomy for the backbone of the ingroup.

## Discussion

The genus *Krogia* was first described by Timdal in 2002 and only few reports of the genus have been published since (Lumbsch et al. 2011; Timdal 2009). Furthermore, no molecular phylogenetic studies investigating the monophyly of this genus have been conducted. In our study, we present the first multi-locus phylogenetic hypothesis of the genus *Krogia* (Fig. 1) and describe three new species from the Paleotropics based on molecular, morphological and chemical data.

All accessions of *Krogia* included in our molecular phylogeny (Table 1) form a well-supported, monophyletic group (Fig. 1). Five strongly supported clades can be distinguished within the genus. These five clades are delimited by rather long branches in comparison to the short terminal branches, indicating that the five clades correspond to five different species (Fig. 1). Two clades correspond to the two previously described species *K.coralloides* (Timdal 2002) and *K.antillarum* (Timdal 2009), while the remaining three clades correspond to the three new species *K.borneensis*, *K.isidiata* and *K.macrophylla*. The new species are morphologically distinct from one another and from the three known species, *K.antillarum*, *K.coralloides* and *K.microphylla*: *Krogiaborneensis* forms more elongated and often linear squamules, *K.isidiata* forms characteristically long and sparingly branched isidia and *K.macrophylla* is a large species with wider squamules than any of the known species. We therefore describe them as new species. All *Krogia* species known contain the characteristic red or purple spots on the thallus and apothecia, consisting of one or more unknown pigments.

Our specimens of the genus *Krogia* were typically found amongst collections of undetermined tropical rainforest lichens, particularly amongst those tentatively named *Phyllopsora*. Timdal (2002) suggested a close relationship between *Krogia* and *Phyllopsora* based on overall morphological similarity. The two genera are anatomically distinct (Timdal 2002), although both form small squamules or lobes on bark. A comprehensive molecular phylogeny of the family Ramalinaceae, however, revealed the type species of the two genera to belong to different major clades within the family (Kistenich et al. in press). They are therefore not as closely related as previously anticipated.

On detailed microscopic examination of specimens of the new species *K.borneensis*, we discovered a thin, unicellular cortex on the upper and lower side of the thallus. This type of cortex, with a single layer of rounded or cuboid cells and a thick cell wall, is characteristic for the tropical genus *Eschatogonia* Trevis. (Timdal 2008). The cellular cortex surrounding the fungal tissue in *K.borneensis* has thinner cell walls and consists of somewhat longer, rather rectangular cells instead of the round and cuboid cells observed in *Eschatogonia* species. Our molecular phylogenetic hypothesis confirms that *Krogia* is not closely related to *Eschatogonia*. This indicates that the characteristic cortex in *Eschatogonia* has evolved independently.

*Krogia* is resolved as the phylogenetic sister to a clade consisting of the type species of *Bacidina*, *B.phacodes* (Körb.) Vězda and *B.brittoniana* (Riddle) LaGreca & Ekman (Fig. 1). *Krogia* differs from *Bacidina* s.str. (sensu Kistenich et al. in press) in having spirally arranged ascospores and a non- to weakly amyloid ascus tholus.

In recent years, lichenologists have increasingly focused on tropical regions and many new species have been described each year (e.g. Aptroot et al. 2018; Lücking et al. 2014; Masson et al. 2015; Naksuwankul et al. 2016; Sodamuk et al. 2017). It seems that the full diversity of tropical lichens is yet to be discovered. In our study, we report two new species of *Krogia*, *K.isidiata* and *K.macrophylla*, from but one island, the island Grande Terre belonging to New Caledonia. Therefore, further extensive collecting expeditions to remote tropical areas are necessary to explore the total diversity of the genus *Krogia*.

## Taxonomy

### Krogia borneensis

Taxon classificationFungiLecanoralesRamalinaceae

Kistenich & Timdal sp. nov.

MB825078

Fig. 2

#### Diagnosis.

The species differs from *K.isidiata* in forming lacinules as vegetative dispersal units, not isidia, and from the other species in the genus in producing sekikaic and homosekikaic acid.

#### Type.

Malaysia, Borneo, Sabah, Maliau conservation area, trail between Nepenthes Camp and waterfall Takob Akob, 4°43.4'N, 116°52.2'E, 900–1000 m alt., in low (few metres) and open pristine montane "Kerangas" (heath) forest with higher trees mostly along a small stream, on smooth barked tree in the vicinity of the stream, 2013-02-23, C. Vairappan & H. Thüs L291 (BORH, holotype) [TLC: sekikaic and homosekikaic acid; GenBank: MH174275 (mtSSU)].

#### Description.

Thallus effuse, squamulose; squamules up to 1 mm wide, deeply divided into 0.1–0.2 wide lobes, ascending, imbricate, flattened, elongated to partly linear, often slightly laterally constricted, greyish-green with patches of red (K+ purple) spots, epruinose, glabrous; margin concolorous with upper side, not fibrillose; lower side white; lacinules formed by tips of the lobes. Upper cortex composed of a single layer of thick-walled cells with angular to shortly cylindrical lumina (resembling *Eschatogonia*-type), not containing crystals (polarised light!); algal layer 30–40 µm thick, filled with crystals dissolving in K; medulla composed of loosely interwoven hyphae, not containing crystals dissolving in K; lower cortex resembling upper cortex, both continuing over the edge of the squamule; prothallus brownish-black, often well developed. Apothecia (present in the holotype only) up to 0.6 mm diam. when simple, forming aggregates up to 1.5 mm diam., medium brown with red patches or entirely reddish-brown, more or less plane, with an indistinct, slightly paler, often flexuose margin; excipulum pale brown to colourless, composed of radiating, closely conglutinated hyphae, in inner part containing colourless crystals dissolving in K; hypothecium partly to entirely stained by a blood red pigment which dissolves in K with a purple effusion; epithecium colourless, not containing crystals. Ascospores filiform, curved, non-septate, spirally arranged in ascus, 20–31 × ca. 1.0 µm (n=10, from holotype). Conidiomata not seen.

#### Chemistry.

Sekikaic acid (major), homosekikaic acid (major). Spot tests: all negative, except for red patches being K+ purple.

#### Distribution.

The species is known from five localities in Borneo.

**Figure 2.**
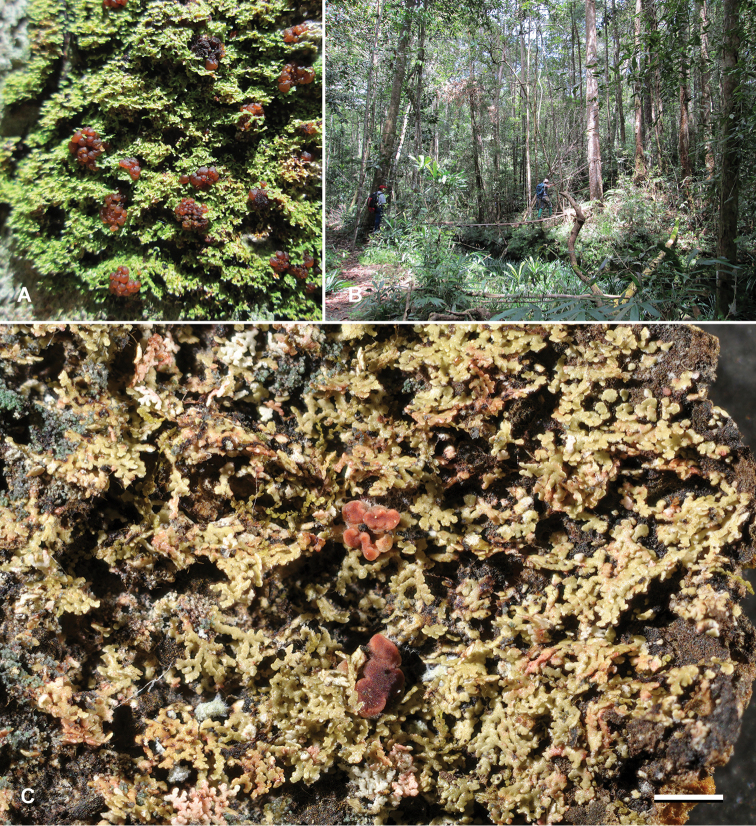
*Krogiaborneensis*. **A** Field photograph of the holotype **B** habitat at type locality **C** herbarium photograph of holotype. Scale bar: 1 mm. Photo: H. Thüs (**A, B**), E. Timdal (**C**).

**Ecology**. The species occurred in rather low "Kerangas" (heath) forest vegetation or on transition vegetation between the heath and oak/conifer (particularly *Agathis*) forest at higher elevations (ca. 1000 m) on very poor soils on sandstone (Fig. 2B). The species always grew on the rather smooth barked, middle-sized trees together with various Pyrenulaceae and Graphidaceae.

#### Etymology.

The specific epithet refers to its occurrence in Borneo.

#### Remarks.

The medium-sized, flattened squamules make the species morphologically most similar to the neotropical *K.antillarum*. The squamules are more elongated, often linear and with more lateral constrictions in *K.borneensis* than in *K.antillarum*, which has more fan-shaped squamules. The former species has a thin, unicellular cortex on both upper and lower side, whereas the latter has a multicellular (20–30 µm thick) upper cortex and lacks a lower cortex (Timdal 2009). Chemically, the latter species differs in forming 4-O-methylcryptochlorophaeic acid.

*Krogiaisidiata* shares the secondary chemistry (sekikaic and homosekikaic acid) with *K.borneensis*, but they differ in their vegetative dispersal units, the former producing cylindrical isidia, the latter flat lacinules (fragmenting squamules). The upper cortex of *K.isidiata* is multicellular (15–30 µm thick) and the lower cortex is absent.

#### Additional specimens examined.

Malaysia, Borneo. *Sabah*: Danum, plot 88, dipterocarp forest logged in 1988, 4°58'N, 117°50'E, 131 m alt., 1997-04-30, P. Wolseley Q21 p.p. (BM 001104020); Danum valley, pristine lowland dipterocarp forest 4°57.96'N, 117°47.32'E, 200–400 m alt., 2012, P. Wolseley, H. Thüs & C. Vairappan D-3-10-2 (BORH); Maliau conservation area, trail between Nepenthes Camp and waterfall Takob Akob, transition between pristine montane "Kerangas" (heath forest) and montane oak-conifer (*Agathis*) forest, 4°42.6'N, 116°52.5'E, 900–1000 m alt., 2013, C. Vairappan & H. Thüs L229 (BM); Ranau district, Kinabalu park, Musang camp on the Tambuyukon trail (loc. T98), 6°12.720'N, 116°40.891' E, 1429 m alt., epiphytic, 2014-12-09, A. Paukov 2234 (B).

### Krogia isidiata

Taxon classificationFungiLecanoralesRamalinaceae

Kistenich & Timdal sp. nov.

MB825079

Fig. 3

#### Diagnosis.

The species differs from *K.borneensis* in forming isidia as vegetative dispersal units, not lacinules, and from the other species in the genus in producing sekikaic and homosekikaic acid.

#### Type.

New Caledonia, Province Sud, 20 km NNE of Nouméa, along dirt mountain road to Mt Dzumac, 3–400 m S of Seismic Station, ca. 22°03'S, 166°25'E, 830 m alt., on unidentified tree trunk in forest near the road, 2005-12-06, A. Elvebakk 05:633 (O L-186393, holotype; CANB, isotype [not seen]) [TLC: sekikaic and homosekikaic acid; GenBank: MH174285 (ITS)].

#### Description.

Thallus effuse, squamulose; squamules up to 0.4 mm wide, rounded and adnate when young, later becoming somewhat elongated with a crenulate and slightly ascending margin, flattened, green, with scattered patches of red (K+ purple) spots, epruinose, glabrous; margin concolorous with upper side, not fibrillose; lower side white; isidia attached marginally to the squamules, simple or sparingly branched, up to 1.8 mm long and 0.1 mm wide. Upper cortex composed of a few layers of thick-walled, irregularly or mainly periclinally orientated hyphae with angular to shortly cylindrical lumina, 15–30 µm thick, lacking an epinecral layer, not containing crystals (polarised light!); algal layer 30–40 µm thick, filled with crystals dissolving in K; medulla composed of loosely interwoven hyphae, containing crystals in the upper part; lower cortex lacking; prothallus brownish-black, well developed. Apothecia up to 0.8 mm diam. when simple, often forming aggregates up to 1.6 mm diam., dark reddish-brown to brownish-black, more or less plane, with a rather distinct, concolorous or slightly darker, flexuose margin; excipulum dark reddish-brown throughout, composed of radiating, closely conglutinated, thick-walled hyphae with narrowly cylindrical lumina, inner part containing crystals dissolving in K; hypothecium dark reddish-brown, composed of closely conglutinated, thick-walled hyphae with narrowly cylindrical lumina, containing crystals dissolving in K; epithecium colourless, not containing crystals (but crystals present in hymenium below). Ascospores filiform, curved, simple, spirally arranged in ascus, ca. 20–30 × ca. 1.0 µm (estimate of curved spores). Conidiomata not seen.

**Figure 3.**
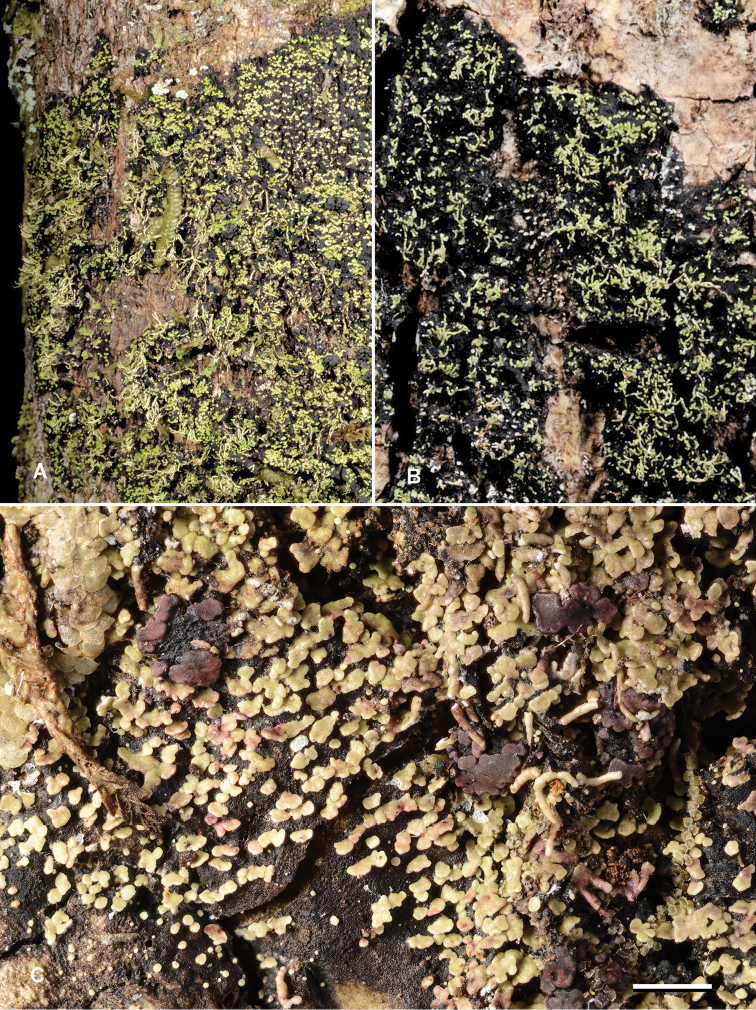
*Krogiaisidiata.***A** field photograph of JR35688**B** field photograph of JR35034**C** herbarium photograph of holotype. Scale bar: 1 mm. Photo: J. Rikkinen (**A, B**), E. Timdal (**C**).

#### Chemistry.

Sekikaic acid (major), homosekikaic acid (major). Spot tests: all negative, except for red patches being K+ purple.

#### Distribution.

The species is known from four collections at three localities in New Caledonia.

**Ecology**. The species grows on tree trunks in moist or mesic tropical forests and woodlands (Fig. 5B). All collections are from low-elevation sites and from ultramafic soils typical of the southern part of Grande Terre (main island of New Caledonia). It prefers shaded basal trunks that are otherwise mainly dominated by epiphytic bryophytes and/or leprarioid lichens.

#### Etymology.

The specific epithet refers to its vegetative dispersal units, isidia.

#### Remarks.

This species and *K.macrophylla* are the only isidiate species of *Krogia*. They differ morphologically mainly in the size and shape of the squamules. In *K.isidiata*, they are small (up to 0.4 mm wide), rounded and adnate to somewhat elongated and with a slightly ascending margin and, in *K.macrophylla*, large (up to 3 mm wide), elongated and ascending even when young. In *K.isidiata*, the squamules are attached to a prothallus, whereas in the latter species, a prothallus has not been observed. The former species contains sekikaic and homosekikaic acid, the latter an unknown compound resembling boninic acid.

*Krogiaisidiata* shares the secondary chemistry with *K.borneensis*; see that species for discussion.

#### Additional specimens examined.

New Caledonia. *Province Sud*: Yaté, dense forests along road RP 3 about 5 km west of Yaté, on tree trunk, 22°10'03.63"S, 166°54'10.15"E, 410 m alt., 2016-09-20, J. Rikkinen 34385 (H); Blue River Provincial Park, dense riparian forest near camp site on river bank, on tree trunk, 22°05'54.79"S, 166°38'20.24"E, 200 m alt. 2016-09-22, J. Rikkinen 35034 (H); Blue River Provincial Park, dense forest between camp site and road GR NC1, on tree trunk, 22°05'47.63"S, 166°38'22.54"E, 220 m alt., 2016-09-24, J. Rikkinen 35688 (H, PC).

### Krogia macrophylla

Taxon classificationFungiLecanoralesRamalinaceae

Kistenich & Timdal sp. nov.

MB825080

Fig. 4

#### Diagnosis.

The species differs from all other species of the genus in forming larger (up to 3 mm wide, vs. up to 0.3–1.5 mm wide in the other species) squamules and, except for *K.coralloides*, in producing an unknown compound resembling boninic acid.

#### Type.

New Caledonia, Province Sud, Mont Mou Nature Reserve, in low dense mist forest along foot path to the mountain summit, on tree trunk, 22°03'39.66"S, 166°20'53.54"E, 1162 m alt., 2016-09-26, J. Rikkinen 36077 (H, holotype [TLC: unknown compound resembling boninic acid; GenBank: MH174288 (ITS)]; PC, isotype).

#### Description.

Thallus effuse, squamulose; squamules up to 3 mm wide, at first rounded, later becoming incised and deeply divided into up to 1 mm wide lobes, ascending even when young, often imbricate, flattened or with an up-turned tip, greyish-green, with patches of purple (K+ bluish-black) spots, epruinose, glabrous; margin concolorous with upper side, not fibrillose; lower side white; isidia (present in one specimen) attached marginally to the squamules, simple or sparingly branched, up to 1.6 mm long and 0.2 mm wide. Upper cortex composed of thick-walled, irregularly orientated hyphae with angular to cylindrical lumina, 50–80 µm thick, lacking an epinecral layer, not containing crystals (polarised light!); algal layer 25–35 µm thick, filled with crystals dissolving in K; medulla composed of loosely interwoven hyphae, upper part containing crystals dissolving in K; lower cortex lacking; prothallus lacking. Apothecia up to 1 mm diam. when simple, often forming aggregates up to 6 mm diam., pale to medium brown, with purple patches, plane to weakly convex, with an indistinct, slightly paler, often flexuose margin; excipulum pale brown to colourless, composed of radiating, closely conglutinated, thick-walled hyphae with narrowly cylindrical lumina, not containing crystals; hypothecium pale brown to colourless, composed of closely conglutinated, thick-walled hyphae with narrowly cylindrical lumina, not containing crystals; epithecium colourless, not containing crystals; purple pigment occurring patchily in exciple, hypothecium and hymenium. Ascospores filiform, curved, simple, spirally arranged in ascus, ca. 20–30 × ca. 1.0 µm (estimate of curved spores). Conidiomata not seen.

**Figure 4.**
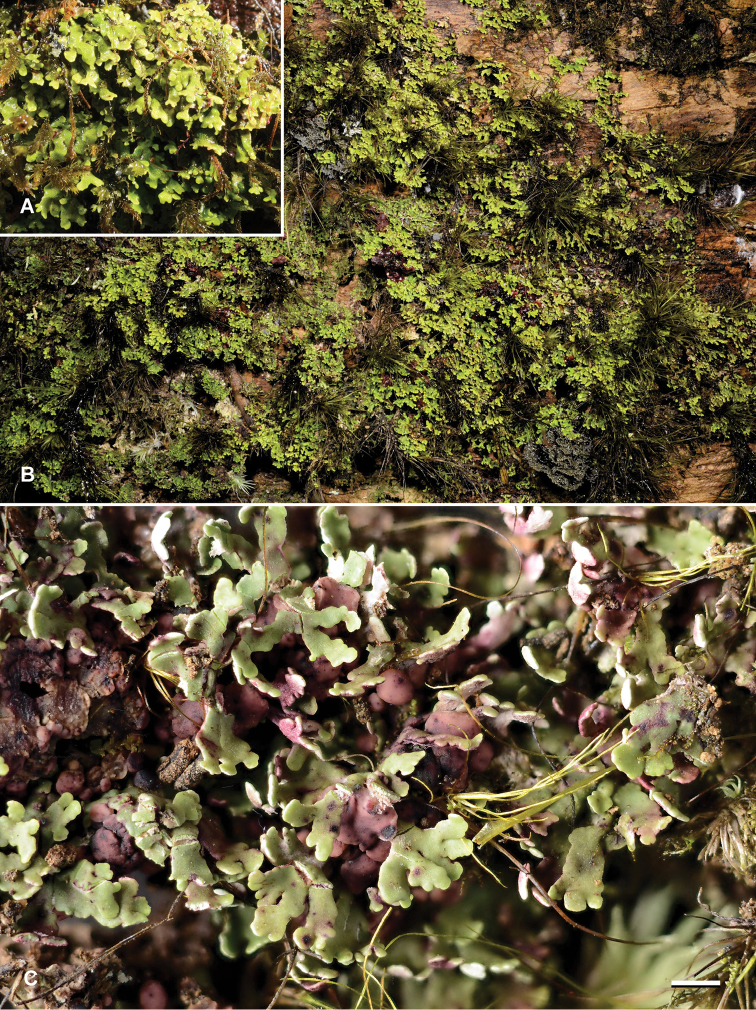
*Krogiamacrophylla***A** field photograph of JR36047**B** field photograph of holotype **C** herbarium photograph of holotype. Scale bar: 1 mm. Photo: J. Rikkinen (**A, B**), E. Timdal (**C**).

#### Chemistry.

An unknown compound resembling boninic acid (major) and traces of additional compounds. Spot tests: all negative, except for purple patches being K+ deeper purple to bluish-black.

#### Distribution.

The species is known from three localities in New Caledonia.

#### Ecology.

**Figure 5.**
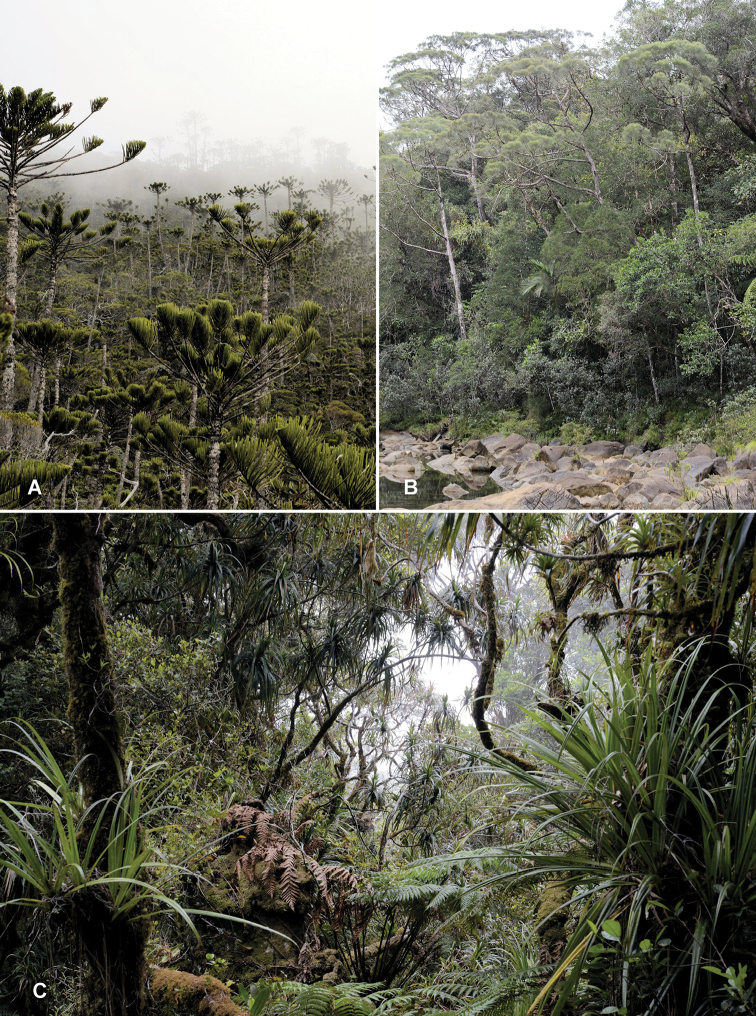
Habitat images from New Caledonia **A** Mont Humboldt Nature Reserve, site of *K.macrophylla*, with *Araucariahumboldtensis***B** Blue River Provincial Park, site of *K.isidiata* and *K.macrophylla***C** Mont Mou Nature Reserve, holotype locality of *K.macrophylla*. Photo: J. Rikkinen.

The species grows on tree trunks in moist or wet tropical forests (Figs 5A–C). Two collections are from montane mist forests and one from a low-elevation rainforest, all on ultramafic soils typical of the southern part of Grande Terre (main island of New Caledonia). It prefers shaded basal trunks that are otherwise mainly dominated by epiphytic bryophytes and/or leprarioid lichens.

#### Etymology.

The specific epithet refers to the large squamules.

#### Remarks.

In the examined material, one specimen (Rikkinen 38565) is isidiate, whereas the others are not. Our first assumption, that two species were involved, was not confirmed by the phylogeny (Fig. 1) and it appears that vegetative dispersal units, isidia, are produced occasionally in *K.macrophylla*. The only other isidiate species of *Krogia* is *K.isidiata*; see that species discussion.

*Krogiamacrophylla* has a similar secondary chemistry to *K.coralloides* (an unknown substance resembling boninic acid as the major constituent) but differs in lacking the boninic acid that co-occurs as the major constituent in *K.coralloides* (Timdal 2002). *Krogiacoralloides* forms smaller (up to 1 mm wide), more linear lobes with often down-turned tips.

#### Additional specimens examined.

New Caledonia. *Province Sud*: Blue River Provincial Park, dense riparian forest near camp site on river bank, on tree trunk, 22°05'54.79"S, 166°38'20.24"E, 200 m alt., 2016-09- 22, J. Rikkinen 35037 (H); locality data as for holotype, J. Rikkinen 36047 (H); Mont Humboldt Nature Reserve, close to Mont Humboldt refuge, in low dense mist forest along foot path from shelter towards the mountain summit, on tree trunk, 21°52'46.79"S, 166°24'49.17"E, 1320 m alt., 2011-11-09, J. Rikkinen 38565 (H).

##### Key to the species of *Krogia*

**Table:** 

1	Squamules large, up to 3 mm wide and with up to 1 mm wide lobes; containing an unknown compound resembling boninic acid	*** K. macrophylla ***
–	Squamules smaller, up to 1.5 mm wide and with up to 0.4 mm wide lobes; chemistry various	**2**
2	Thallus with isidia; containing sekikaic and homosekikaic acid	*** K. isidiata ***
–	Thallus without isidia; chemistry various	**3**
3	Squamules minute, up to 0.3 wide and with up to 0.1 mm, simple lobes, forming a microphyllinous crust; not containing lichen substances	*** K. microphylla ***
–	Squamules medium sized, up to 1.5 mm wide and with up to 0.4 mm wide, coralloid elongated lobes; containing lichen substances	**4**
4	Thallus with brownish black hypothallus; containing sekikaic and homosekikaic acid	*** K. borneensis ***
–	Thallus without distinct hypothallus; chemistry different	**5**
5	Squamules mainly flattened; lobes up to 0.4 mm wide; containing 4-O-methylcryptochlorophaeic acid	*** K. antillarum ***
–	Squamules mainly convex; lobes up to 0.1 mm wide; containing boninic acid and an unknown, similar compound	*** K. coralloides ***

## Supplementary Material

XML Treatment for Krogia borneensis

XML Treatment for Krogia isidiata

XML Treatment for Krogia macrophylla

## Appendix

*Krogiaantillarum* is reported here as new to Brazil, Guatemala and Mexico from the following examined specimens: Brazil. *Rio de Janeiro*: Parque Nacional do Itatiaia, along trail to Três Picos, 22.4358°S, 44.6118°W, 1090 m alt., on tree trunk in Atlantic rainforest, 2015-11-27, M.S. Dahl, S. Kistenich, E. Timdal & A.K. Toreskaas AM-39 (O L-202829). Guatemala. *Depto. Alta Verapaz*: NE of Cobán-Aragon, at the borders of Rio Cahabon (tierra fría), 1700 m alt., cloud forest, on *Liquidambartyraciflua*, 2002-09-13, C. Andersohn s.n. (B 60-127330. Mexico. *Chiapas*: Municipio La Trinitaria, Parque Nacional Lagunas de Montebello, Paso del Soldado, 16°07'07"N, 91°43'09"W, 1500 m alt., bosque de *Pinusmaximinoi* y *Quercussapotifolia*, exposición N, epífita, 1994-11-29, J. Wolf & H. Sipman 2052 (B 60-110597).

## References

[B1] Altekar G Dwarkadas S Huelsenbeck JP Ronquist F (2004) Parallel metropolis coupled Markov chain Monte Carlo for Bayesian phylogenetic inference. Bioinformatics 20: 407–415. 14960467 10.1093/bioinformatics/btg427

[B2] Aptroot A Sipman HJM Mercado Diaz JA Mendonça CO Feuerstein SC Cunha-Dias IPR Pereira TA Cáceres MES (2018) Eight new species of Pyrenulaceae from the Neotropics, with a key to 3-septate *Pyrgillus* species. The Lichenologist 50: 77–87. 10.1017/S0024282917000573

[B3] Ayres DL Darling A Zwickl DJ Beerli P Holder MT Lewis PO Huelsenbeck JP Ronquist F Swofford DL Cummings MP Rambaut A Suchard MA (2012) BEAGLE: An Application Programming Interface and High-Performance Computing Library for Statistical Phylogenetics. Systematic Biology 61: 170–173. 10.1093/sysbio/syr100 21963610 PMC3243739

[B4] Bendiksby M Timdal E (2013) Molecular phylogenetics and taxonomy of *Hypocenomyce* sensu lato (Ascomycota: Lecanoromycetes): Extreme polyphyly and morphological/ecological convergence. Taxon 62: 940–956. 10.12705/625.18

[B5] Culberson CF (1972) Improved conditions and new data for the identification of lichen products by a standardized thin-layer chromatographic method. Journal of Chromatography 72: 113–125. 10.1016/0021-9673(72)80013-X 5072880

[B6] Culberson CF Johnson A (1982) Substitution of methyl tert.-butyl ether for diethyl ether in standarzided thin-layer chromatographic method for lichen products. Journal of Chromatography 238: 483–487. 10.1016/S0021-9673(00)81336-9

[B7] Gardes M Bruns TD (1993) ITS primers with enhanced specificity for basidiomycetes – application to the identification of mycorrhizae and rusts. Molecular Ecology 2: 113–118. 8180733 10.1111/j.1365-294x.1993.tb00005.x

[B8] Katoh K Standley DM (2013) MAFFT Multiple Sequence Alignment Software Version 7: Improvements in Performance and Usability. Molecular Biology and Evolution 30: 772–780. 10.1093/molbev/mst010 23329690 PMC3603318

[B9] Kearse M Moir R Wilson A Stones-Havas S Cheung M Sturrock S Buxton S Cooper A Markowitz S Duran C (2012) Geneious Basic: an integrated and extendable desktop software platform for the organization and analysis of sequence data. Bioinformatics 28: 1647–1649. 22543367 10.1093/bioinformatics/bts199 PMC3371832

[B10] Kistenich S Timdal E Bendiksby M Ekman S (in press) Molecular systematics and character evolution in the lichen family Ramalinaceae (Lecanorales, Ascomycota). Taxon 67. 10.12705/675.1

[B11] Lanfear R Frandsen PB Wright AM Senfeld T Calcott B (2016) PartitionFinder 2: new methods for selecting partitioned models of evolution for molecular and morphological phylogenetic analyses. Molecular Biology and Evolution 34: 772–773. 10.1093/molbev/msw260 28013191

[B12] Lücking R Johnston MK Aptroot A Kraichak E Lendemer JC Boonpragob K Cáceres ME Ertz D Ferraro LI Jia Z-f (2014) One hundred and seventy-five new species of Graphidaceae: closing the gap or a drop in the bucket? Phytotaxa 189: 7–38.

[B13] Lumbsch HT Chaves-Chaves JL Umaña-Tenorio L Lücking R (2011) One hundred new species of lichenized fungi: A signature of undiscovered global diversity. Phytotaxa: 1–127.

[B14] Masson D Divakar PK Sérusiaux E (2015) *Hypotrachynapenduliloba* and *Remototrachynapandani*, two new species in the hyperdiverse lichen family Parmeliaceae from Réunion in the Mascarene Archipelago. Mycological Progress 14: 1–15.

[B15] Menlove JE (1974) Thin-layer chromatography for the identification of lichen products. Bulletin of the British Lichen Society 34: 3–5.

[B16] Miller MA Pfeiffer W Schwartz T (2010) Creating the CIPRES Science Gateway for inference of large phylogenetic trees. Proceedings of the Gateway Computing Environments Workshop (GCE), 2010, New Orleans, LA, 1–8.

[B17] Mirarab S Nguyen N Guo S Wang L-S Kim J Warnow T (2015) PASTA: ultra-large multiple sequence alignment for nucleotide and amino-acid sequences. Journal of Computational Biology 22: 377–386. 25549288 10.1089/cmb.2014.0156 PMC4424971

[B18] Naksuwankul K Kraichak E Parnmen S Lücking R Lumbsch H (2016) Five new species of Graphidaceae (Ascomycota, Ostropales) from Thailand. MycoKeys 17: 47–63.

[B19] Rannala B Zhu T Yang Z (2011) Tail paradox, partial identifiability, and influential priors in Bayesian branch length inference. Molecular Biology and Evolution 29: 325–335. 21890479 10.1093/molbev/msr210

[B20] Ronquist F Huelsenbeck JP (2003) MrBayes 3: Bayesian phylogenetic inference under mixed models. Bioinformatics 19: 1572–1574. 12912839 10.1093/bioinformatics/btg180

[B21] Sodamuk M Boonpragob K Mongkolsuk P Tehler A Leavitt SD Lumbsch HT (2017) *Kalbionorapalaeotropica*, a new genus and species from coastal forests in Southeast Asia and Australia (Malmideaceae, Ascomycota). MycoKeys 22: 15–25.

[B22] Stamatakis A (2014) RAxML version 8: a tool for phylogenetic analysis and post-analysis of large phylogenies. Bioinformatics 30: 1312–1313. 24451623 10.1093/bioinformatics/btu033 PMC3998144

[B23] Stöver BC Müller KF (2010) TreeGraph 2: Combining and visualizing evidence from different phylogenetic analyses. BMC Bioinformatics 11: 7. 10.1186/1471-2105-11-7 PMC2806359 20051126

[B24] Timdal E (2002) *Krogiacoralloides*, a new lichen genus and species from Mauritius. The Lichenologist 34: 293–296.

[B25] Timdal E (2008) Studies on *Eschatogonia* (Ramalinaceae) in Peru. Lichenologist 40: 31–38.

[B26] Timdal E (2009) *Krogiaantillarum* (Ramalinaceae), a new lichen species from the West Indies. Bryologist 112: 387–389.

[B27] White TJ Bruns T Lee S Taylor J (1990) Amplification and direct sequencing of fungal ribosomal RNA genes for phylogenetics. PCR protocols: a guide to methods and applications, 18: 315–322.

[B28] Zhang C Rannala B Yang Z (2012) Robustness of Compound Dirichlet Priors for Bayesian Inference of Branch Lengths. Systematic Biology 61: 779–784. 10.1093/sysbio/sys030 22328570

[B29] Zoller S Scheidegger C Sperisen C (1999) PCR primers for the amplification of mitochondrial small subunit ribosomal DNA of lichen-forming ascomycetes. The Lichenologist 31: 511–516. 10.1006/lich.1999.0220

[B30] Zwickl DJ (2006) Genetic algorithm approaches for the phylogenetic analysis of large biological sequence datasets under the maximum likelihood criterion. PhD Dissertation, University of Texas at Austin, Austin, Texas.

